# Case Report: Case report: Rapidly progressing tooth wear dominated by intrinsic and extrinsic erosion

**DOI:** 10.12688/f1000research.142183.2

**Published:** 2024-03-05

**Authors:** Shivaughn Marchan, Estévan Ollivierra, Alexa Diaz, Rochard Santo

**Affiliations:** 1 The University of the West Indies at St Augustine, Saint Augustine, Tunapuna/Piarco Municipal Corporation, Trinidad and Tobago

**Keywords:** Tooth wear, erosion, gastroesophageal reflux disease, diet

## Abstract

Tooth wear is multi-factorial presenting as a combination of abrasion, attrition, and erosion. This case report represents a case of combined tooth wear in a 46-year-old Indo-Trinidadian male, with a predominant erosive component with both the clinical signs and features of intrinsic and extrinsic erosion. This patient case is unique since the wear predominated by dental erosion has occurred rapidly evidenced by the physical clinical appearance of a lack of compensation and the upper left premolars and molars relatively unaffected by the overall effects of tooth wear. This lack of compensation, where opposing teeth have not supra-erupted to maintain inter-arch stability, and the maintenance of occlusal vertical dimension on the left due to the non-worn posterior maxillary teeth, provides the benefit of simplifying subsequent restorative management.

The medical and diet history corroborates the diagnoses of intrinsic and extrinsic erosion respectively. Complications noted with rapid tooth wear, such as dentine sensitivity and pulpal necrosis are known sequelae of tooth wear however the patient presented in this case report shows a concomitant high caries experience and poor oral hygiene. Cases such as the one presented here require not only comprehensive dental management, utilizing a restorative approach but also medical referral for confirmation of a diagnosis and management of gastroesophageal reflux disease. Inherent to the management of this patient should be a multidisciplinary medical and dental approach, with confirmation and management of the cause of the intrinsic erosion as well as restorative dental management, together with dietary counseling to mitigate the effect of intrinsic and extrinsic sources of acid on dental hard tissue. A key lesson learned from this case is the importance of history and targeted questioning when trying to determine the cause of tooth wear dominated by intrinsic and extrinsic erosion.

## Introduction

Tooth wear or tooth surface loss is defined as the irreversible loss or degeneration of hard dental tissue caused by non-bacterial means. ^1^ 20% of tooth wear is multifactorial, presenting clinically as a combination of dental erosion, attrition, and abrasion. ^1^ Research on a Trinidadian patient sample attending a dental teaching hospital concluded that 72% of the studied sample suffered from mild, moderate, or severe wear, with 52% exhibiting mild wear. ^2^ Erosive wear, which causes a softening of tooth enamel by endogenous or exogenous acids can exacerbate and accelerate wear caused by attrition and abrasion with clinical signs of chipping or fracture of incisal edges or hard tissue loss at the cervical margins of teeth respectively. ^3^ Age and sex are determinants in the prevalence and severity of erosive tooth wear. ^4^ Erosion of dental tissue can be further diagnosed as intrinsic or extrinsic erosion depending on the main source of acid causing tooth wear and the clinical pattern of wear. ^3^ Intrinsic wear, clinically evident as cupped-out occlusal lesions or affecting the palatal surface of upper anterior teeth, is normally associated with medical conditions such as gastroesophageal reflux disease (GERD), chronic vomiting associated with pregnancy, alcoholism, or eating disorders. ^3^ Conversely, extrinsic erosion normally affects facial or buccal surfaces and is commonly associated with exogenous acids found in the consumption of citrus fruits and a variety of beverages. ^5^ Additionally, a “swish and swallow” pattern of beverage consumption can exacerbate extrinsic erosion with this pattern of beverage consumption causing erosive lesions on the palatal surfaces of upper teeth. ^6^

## Case presentation

**Figure 1.**
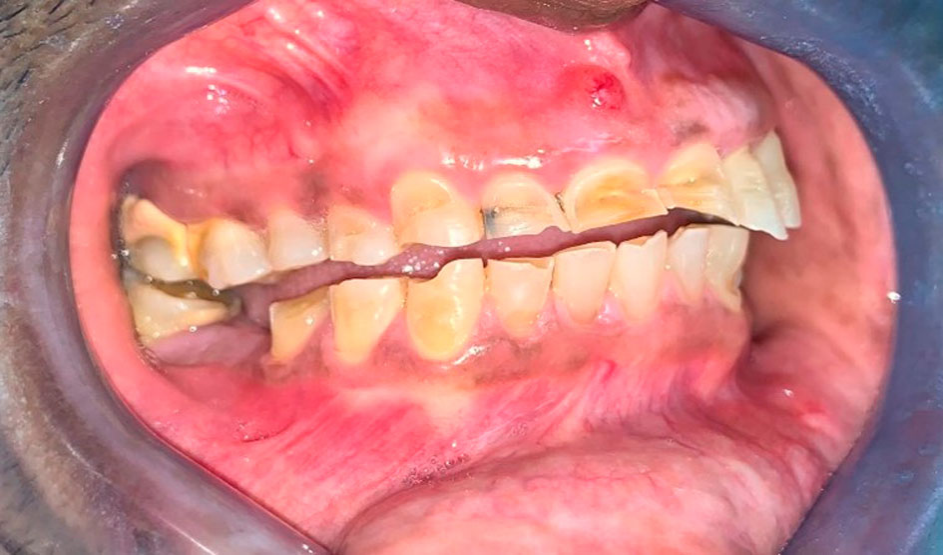
Right buccal view of the worn teeth showing lack of occlusal compensation.

This case report was prepared using the CARE guidelines described by Gagnier *et al.* which provides a 13-item checklist to improve reporting of case reports in the medical literature. ^7^ A 46-year-old Indo-Trinidadian male agriculturist, of South Asian descent, presented to a clinic of a dental teaching hospital with two main complaints of pain associated with tooth 1.1 and his enamel wearing away on several teeth, which started only three years previously ( Figure 1). The patient’s medical history included frequent consumption of citrus fruits, particularly oranges and grapefruits where the anterior teeth would be embedded into a cut face of the peeled fruit as part of his job. Specifically, he was a budder and grafter of citrus plants at a government-run nursery and often tasted citrus fruit to determine the quality and taste profile of grafted citrus fruit. The patient’s history also revealed frequent consumption of a local delicacy of fresh pickled green fruit referred to as “chow”. The patient suspected undiagnosed GERD, which he referred to as heartburn, and was currently self-medicating with various dosages of omeprazole, between 20mg and 40mg daily, on an intermittent basis when the symptoms of heartburn presented. The patient admitted to social drinking on the weekends mainly with the consumption of Pilsner beer which he believed exacerbated his heartburn. When asked about vomiting after his beer consumption, he noted this happened only occasionally. On questioning, the patient confirmed no other teeth were painful or sensitive currently, but he has suffered from generalized sensitivity in the past. Tooth 1.1 was tender to vertical percussion and was tender on palpation of the root apex. Clinical examination revealed a fistula on the attached mucosa at the apex of this tooth. A periapical radiograph was taken using a paralleling technique. An intra-oral X-ray tube (Carestream. Kodak) using 65KVp and 20mAs was used to expose a size 2 periapical phosphor plate sensor (Carestream, Kodak). This periapical radiograph confirmed a periradicular radiolucency consistent with a pulpal diagnosis of necrosis and chronic periradicular periodontitis.

### Other non-tooth wear clinical findings/diagnoses

Physical clinical examination revealed teeth 3.6, 4.6, and 3.8 were previously extracted. Bitewing radiographs were also taken on the patient’s initial visit, using a Size 2 periapical phosphor plate sensor in a positioning device (XCP, Rinn). The X-ray beam produced by the intra-oral X-ray tube (Carestream, Kodak) was positioned through the posterior tooth contacts a 10-degree vertical angulation. Proximal caries was noted clinically and on bitewing radiographs of several teeth on both the upper and lower arches ( Figure 5). Gross deposits of generalized supra- and sub-gingival calculus were noted. Despite caries being present on both the right and left quadrants, larger carious lesions were noted on the right quadrants where the wear was more severe. Multiple mandibular tori were noted during the clinical examination.

### Clinical presentation of tooth wear lesions

**Figure 2.**
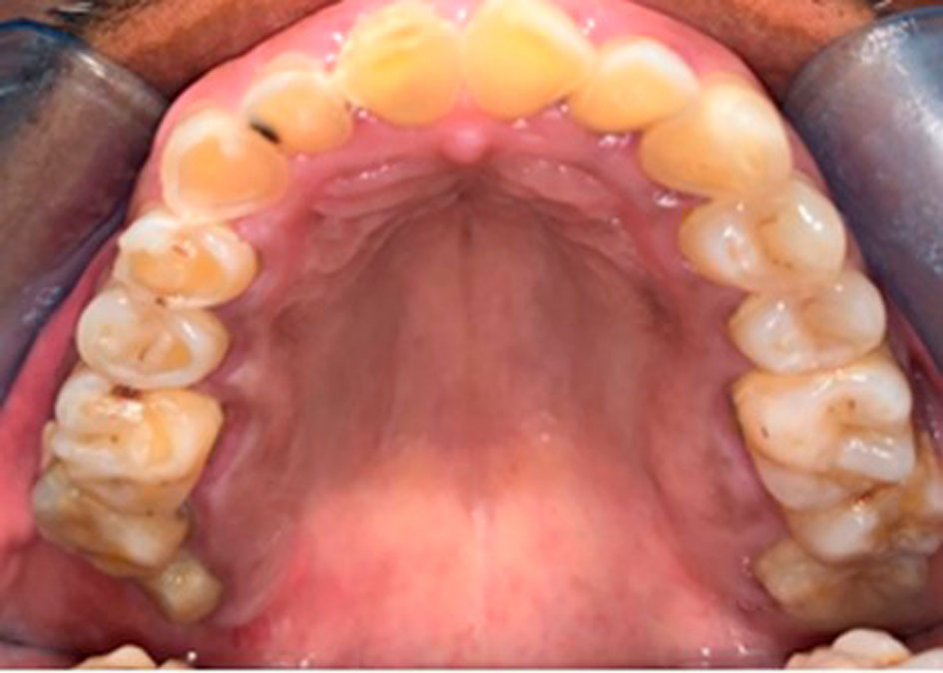
Maxillary view showing pronounced occlusal cupping on the right quadrants.

**Figure 3.**
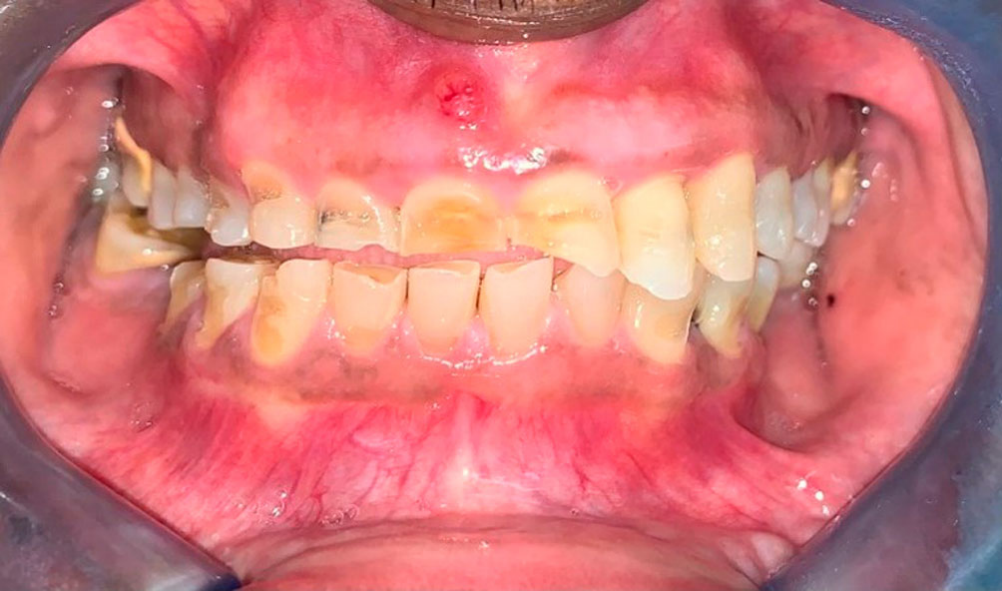
Anterior view of the worn teeth showing facial notching.

**Figure 4.**
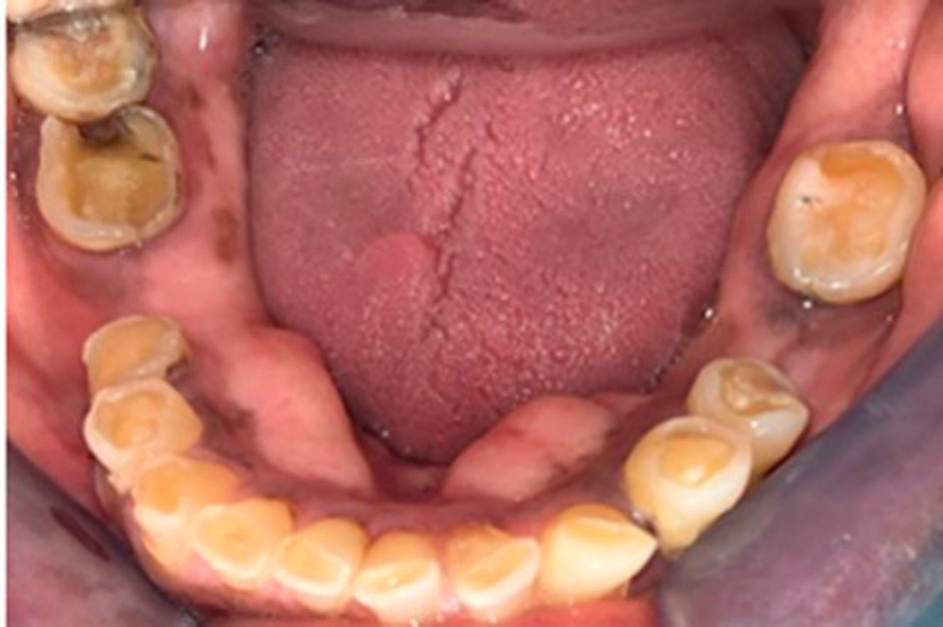
Mandibular view showing pronounced occlusal cupping on all remaining teeth and carious lesions on the lower right.

On physical examination at the time of initial patient presentation, the surfaces of all remaining teeth, except the upper left molars and premolars, showed moderate to severe wear involving both enamel and dentine surfaces. Cupped-out erosive lesions were noted on the occlusal surface of mandibular and maxillary molars and the premolars ( Figure 2, Figure 4). Interestingly the premolars and molars on the upper left side showed only mild occlusal cupping ( Figure 2). Additionally, the palatal surfaces of teeth 6 and 11 showed evidence of intrinsic erosion ( Figure 2). There were moderate to severe erosive lesions involving the cervical to middle thirds of the facial surfaces of teeth 1.4, 1.3, 1.2, 1.1, and 2.1 ( Figure 1). Tooth 8 had the deepest facial lesion involving the entire facial surface of the tooth. These upper teeth had lost coronal tooth height with fractures of the incisal edges. Following this same pattern, milder wear was noted on the facial aspect of teeth 2.2 and 2.3 with two to three striations noted horizontally across the facial surface ( Figure 3). The rate of wear seems to have progressed quickly with no occlusal compensation and an absence of any occlusal contacts between the maxillary and mandibular teeth in the anterior sextants and right quadrants. The inter-occlusal space provided an opportunity to simplify the restorative rehabilitation of the worn teeth. Teeth 4.2, 4.3, 4.4, and 4.5 showed tooth surface loss from the cervical third of these teeth towards the middle third, with tooth 29 having the entire facial surface of the tooth involved ( Figure 1). Cupping was noted on the incisal edges of the lower incisors ( Figure 4).

**Figure 5.**
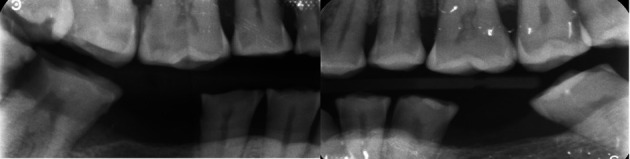
Bitewing radiographs confirming proximal caries.

## Discussion

The patient exhibited wear of multifactorial etiology with clinical signs of attrition, abrasion, and erosion with a predominant erosive component. Of particular concern was a clinical pattern of tooth surface loss that indicated both intrinsic and extrinsic erosion occurring at an accelerated rate evidenced by a lack of compensation and necrosis of the pulp of tooth 1.1. The proposed mechanism of tooth loss from the incisal edges of the upper anterior teeth occurred because of tooth-to-tooth contact on incisal edges which may have been already thinned because of both facial and palatal wear. Tooth brushing of softened dental enamel continuously subjected to both endogenous and exogenous acids could result in abrasive-erosive type lesions noted on the buccal surfaces of the right side. Of note, is the presence of multiple mandibular tori which have been linked to bruxism and occlusal wear. ^8^

Caries is not a normal or prominent feature in the clinical presentation of tooth surface loss of a predominant erosive wear. ^9^ However, loss or slipping of proximal contacts, noted with rapid tooth wear could predispose patients to food packing, plaque trapping, and eventual caries formation. ^10^ This is the working hypothesis in this case where concomitant wear and caries were noted. Alternatively, a change in the composition and properties of the saliva could account for both the rapid erosive tooth wear noted and the concomitant caries experience. ^11^ The poor oral hygiene evidenced by the gross deposits of calculus could have been caused by long periods of dentine sensitivity. ^3^ Chronic dentine sensitivity may lead to less than optimum tooth brushing to prevent an exacerbation of sensitivity.

Presentation of severe clinical tooth surface loss together with the gleaned medical history must signal to the dental clinician a need for multi-disciplinary management of the patient. This patient requires investigation and management by a gastroenterologist since the severity of intrinsic erosion is directly related to contact time with endogenous gastric acid. ^12^ Medical practitioners may deduce the frequency and duration of GERD by assessing tooth surface loss. ^12^ Such investigation is warranted since a recent systematic review concluded there was a constant correlation between dental erosion and GERD. ^13^ Physicians, when suspecting GERD as a differential diagnosis must examine the condition of a patient’s dentition for the signs of dental erosion. ^14^

The patient also requires dietary counseling to reduce the frequency and peculiar habit of citrus consumption. Such counseling can be effective if a relationship between the patient’s presenting complaint of tooth wear and the clinical appearance and pattern of the wear is linked to the type of fruit consumed as well as the peculiar habit of consumption. ^5^ Citrus consumption is positively related to the development of erosive lesions. ^5^^,^^15^ In linking the clinical appearance of the worn teeth, in part, to the dietary consumption of citrus, counseling can be patient-specific and more likely to be adopted as a preventive strategy in curtailing further tooth surface loss. ^15^

### Dental management

Following medical referral and the start of medical and dietary intervention, dental management would include basic periodontal therapy including scaling of teeth and stabilization of active carious teeth, and initiation of root canal treatment on tooth 1.1. Directly placed composite restorations are planned for the severely worn teeth conforming to the patient’s occlusion. ^16^ This restorative strategy can be used given the general maintenance of the patient’s occlusal vertical dimension and lack of compensation providing interocclusal space for sufficient bulk of restorative material while conforming to the existing occlusal scheme. ^3^ Dental management will also involve the use of splint therapy if bruxism is suspected to be contributing to overall tooth wear. Splint therapy can protect directly placed restorations from excessive wear while also protecting tooth structure on the left quadrants. ^3^ Regular reviews including radiographic, visual, and photographic assessments, following dental management, are important to monitor dentition for any further signs of wear and to facilitate ongoing assessment of restorations for repair or replacement. ^3^^,^^17^ Given that this patient presented with several active carious lesions, review at 4-6 monthly intervals is recommended at which time the dentition can be monitored for further signs of tooth wear.

## Ethics and consent

Written informed consent for publication of their clinical details and clinical images and radiographs was obtained from the patient.

## Data Availability

There are no data associated with this article.

## References

[1] Smith B Knight J : A comparison of patterns of tooth wear with aetiological factors. *Br. Dent. J.* 157:16–19. Unit 2. 10.1038/sj.bdj.4805401 6588978

[2] Rafeek R Marchan S Eder A : Tooth surface loss in adult subjects attending a university dental clinic in Trinidad. *Int. Dent. J.* 2006;56(4):181–186. 10.1111/j.1875-595X.2006.tb00092.x 16972391

[3] Eder A Faigenblum M : Tooth Wear: An Authoritative Reference For Dental Professionals and Students: Springer. *Nature.* 2022. 10.1007/978-3-030-86110-0

[4] Stangvaltaite-Mouhat L Pūrienė A Stankeviciene I : Erosive tooth wear among adults in Lithuania: a cross-sectional National Oral Health Study. *Caries Res.* 2020;54(3):283–291. 10.1159/000509872 32937621

[5] Bamise CT Dinyain VE Kolawole KA : Dental erosion due to lime consumption; review of literature and case report. *East Afr. J. Public Health.* 2009;6(2):141–143. 10.4314/eajph.v6i2.51733 20000018

[6] Fusain KS : Erosive potential of drinks consumed in Dubai (Doctoral Dissertation). 2019.

[7] Gagnier JJ Kienle G Altman DG : The CARE guidelines: consensus-based clinical case reporting guideline development. *Glob. Adv. Health. Med.* 2013;2(5):38–43. 10.7453/gahmj.2013.008 24416692 PMC3833570

[8] Steele JG Lamey PJ Sharkey SW : Occlusal abnormalities, pericranial muscle and joint tenderness and tooth wear in a group of migraine patients. *J. Oral. Rehabil.* 1991;18(5):453–458. 10.1111/j.1365-2842.1991.tb01690.x 1800695

[9] Tschammler C Simon A Brockmann K : Erosive tooth wear and caries experience in children and adolescents with obesity. *J. Dent.* 2019;83:77–86. 10.1016/j.jdent.2019.02.005 30825568

[10] Kaidonis JA : Tooth wear: the view of the anthropologist. *Clin. Oral Investig.* 2008;12(Suppl 1):21–26. 10.1007/s00784-007-0154-8 17938977 PMC2563149

[11] Buzalaf MAR Hannas AR Kato MT : Saliva and dental erosion. *J. Appl. Oral Sci.* 2012;20:493–502. 10.1590/S1678-77572012000500001 23138733 PMC3881791

[12] Daley TD Armstrong JE : Oral manifestations of gastrointestinal diseases. *Can. J. Gastroenterol.* 2007;21(4):241–244. 10.1155/2007/952673 17431513 PMC2657699

[13] Picos A Badea ME Dumitrascu DL : Dental erosion in gastroesophageal reflux disease. A systematic review. *Clujul Medical.* 2018;91(4):387–390. 10.15386/cjmed-1017 30564013 PMC6296724

[14] Lee RJ Aminian A Brunton P : Dental complications of gastro-oesophageal reflux disease: guidance for physicians. *Intern. Med. J.* 2017;47(6):619–623. 10.1111/imj.13249 27604164

[15] Chan AS Tran TTK Hsu YH : A systematic review of dietary acids and habits on dental erosion in adolescents. *Int. J. Paediatr. Dent.* 2020;30(6):713–733. 10.1111/ipd.12643 32246790

[16] Milosevic A : Clinical guidance and an evidence-based approach for restoration of the worn dentition by direct composite resin. *Br. Dent. J.* 2018;224(5):301–310. 10.1038/sj.bdj.2018.168 29495026

[17] Boitelle P : Contemporary management of minimal invasive aesthetic treatment of dentition affected by erosion: case report. *BMC Oral Health.* 2019;19(1):123. 10.1186/s12903-019-0807-4 31226976 PMC6587272

