# Supplementary material for: Case Report: Case report: Rapidly progressing tooth wear dominated by intrinsic and extrinsic erosion
Source: F1000Res. 2024 Mar 5;12:1550. Originally published 2023 Dec 1. [Version 2] doi: 10.12688/f1000research.142183.2 (PMC10928413; doi:10.12688/f1000research.142183.2)
Supplement: Peer review history

# Reviewer report: Reviewer response for version 2

**DOI:** 10.5256/f1000research.163161.r252902

**Published:** 2024-03-11

**Authors:** Alex Milosevic

**Affiliations:** 1 Restorative Dentistry, University of Central Lancashire, Preston, England, UK

The authors have revised and improved the manuscript following previous recommendations.

Are enough details provided of any physical examination and diagnostic tests, treatment given and outcomes?

Yes

Is the case presented with sufficient detail to be useful for other practitioners?

Partly

Is sufficient discussion included of the importance of the findings and their relevance to future understanding of disease processes, diagnosis or treatment?

Yes

Is the background of the case’s history and progression described in sufficient detail?

Partly

Reviewer Expertise:

Dental epidemiology & tooth wear research

I confirm that I have read this submission and believe that I have an appropriate level of expertise to confirm that it is of an acceptable scientific standard.

# Reviewer report: Reviewer response for version 1

**DOI:** 10.5256/f1000research.155693.r232109

**Published:** 2024-02-02

**Authors:** Andrew Eder

**Affiliations:** 1 University College London, London, England, UK

This middle-aged Trinidadian patient presented with generalised tooth wear, referred to in some parts of the article as tooth surface loss. Although the tooth wear was reported as being severe for some teeth, the patient had only become aware of the problem over the previous three years. The diagnoses included missing teeth, carious teeth an abscessed upper front tooth and worn teeth with primary erosive and secondary attritive and abrasive aetiologies. Sources for the erosive component were a high work-related fruit intake combined with linking a local fruit delicacy. Compounding this was regular acid intake in the form of beer and also stomach acid reflux, highlighted by 'heartburn' and possibly made worse by the beer. The understanding of the need for a comprehensive assessment and a holistic approach together with the supporting bibliography is what makes this an excellent case presentation. I would, however, suggest that restorative options be expanded in the section on dental management as well as how the preferred option was delivered and then followed up and maintained. As we live longer and keep our teeth for longer, tooth wear is on the increase and this case presentation highlights the breadth of the challenges.

Are enough details provided of any physical examination and diagnostic tests, treatment given and outcomes?

Yes

Is the case presented with sufficient detail to be useful for other practitioners?

Yes

Is sufficient discussion included of the importance of the findings and their relevance to future understanding of disease processes, diagnosis or treatment?

Yes

Is the background of the case’s history and progression described in sufficient detail?

Yes

Reviewer Expertise:

Restorative Dentistry and Prosthodontics - with a focus on managing patients with tooth wear.

I confirm that I have read this submission and believe that I have an appropriate level of expertise to confirm that it is of an acceptable scientific standard.

# Reviewer report: Reviewer response for version 1

**DOI:** 10.5256/f1000research.155693.r232110

**Published:** 2023-12-23

**Authors:** Alex Milosevic

**Affiliations:** 1 Restorative Dentistry, University of Central Lancashire, Preston, England, UK

The authors should state that they followed the CARE guidelines as published by Gagnier JJ et al. 2013 [Ref-1]. The 13-item checklist improves reporting of case reports.

 Introduction

 ‘Research on a Trinidadian population attending a dental teaching hospital concluded that 72% of the studied population suffered from mild, moderate, or severe wear, with 52% exhibiting mild wear.’ Use of population in this context is incorrect as a sample of the population attended the hospital. Better to just say ‘a Trinidadian patient sample’……………….’72% of the sample suffered…’

 ‘Conversely, extrinsic erosion affects facial or buccal surfaces and is commonly associated with exogenous acids found in the consumption of citrus…….’ It is still not universally accepted that intrinsic erosion leads to palatal surface loss and extrinsic leads to labial/facial/buccal tooth surface loss. Extrinsic acid in the form of drinks can result in palatal erosion.

 Case presentation

 ‘complaints of pain associated with tooth 8’ Which tooth is this? Please use FDI notation or describe in full as lower or mandibular right third molar. American & European tooth notation differs & so the authors should use & state FDI which is widely used & recognized.

 'patient’s medical history uncovered'.  Advise substitute *uncovered* with included.

 ‘a *stoma* on the attached mucosa at the apex of this tooth’, stoma may be the local term but sinus or fistula is generally more acceptable

 ‘Physical clinical examination revealed teeth 19, 30, and 17 were previously extracted’. Again please use FDI

 ‘larger carious lesions were noted on the right *quadrants* where the wear was more severe. *Quadrant* not plural

 Clinical presentation of tooth wear lesions.

 ‘the palatal surfaces of teeth 6 and 11 showed evidence of intrinsic erosion (Figure 2). There were moderate to severe erosive lesions involving the cervical to middle thirds of the facial surfaces of teeth 5,6,7,8, and 9 (Figure 1). Tooth 8 had….’  FDI please.

 There is no mention of the mandibular tori as shown in Fig 4. This is of interest as tori have been linked with bruxism and occlusal wear (Steele J., et al.,1991 [Ref-2]).

Are enough details provided of any physical examination and diagnostic tests, treatment given and outcomes?

Yes

Is the case presented with sufficient detail to be useful for other practitioners?

Partly

Is sufficient discussion included of the importance of the findings and their relevance to future understanding of disease processes, diagnosis or treatment?

Yes

Is the background of the case’s history and progression described in sufficient detail?

Partly

Reviewer Expertise:

Dental epidemiology & tooth wear research

I confirm that I have read this submission and believe that I have an appropriate level of expertise to confirm that it is of an acceptable scientific standard, however I have significant reservations, as outlined above.

## References

[1] : The CARE Guidelines: Consensus-based Clinical Case Reporting Guideline Development. *Glob Adv Health Med* .2013;2(5) : 10.7453/gahmj.2013.008 38-43 10.7453/gahmj.2013.008 24416692 PMC3833570

[2] : Occlusal abnormalities, pericranial muscle and joint tenderness and tooth wear in a group of migraine patients. *J Oral Rehabil* .1991;18(5) : 10.1111/j.1365-2842.1991.tb01690.x 453-8 10.1111/j.1365-2842.1991.tb01690.x 1800695
